# Reinforcement Learning-Based Cloud-Aware HAPS Trajectory Optimization in Soft-Switching Hybrid FSO/RF Cooperative Transmission System

**DOI:** 10.3390/s26030948

**Published:** 2026-02-02

**Authors:** Beibei Cui, Shanyong Cai, Liqian Wang, Zhiguo Zhang, Feng Wang

**Affiliations:** 1 School of Electronic Engineering, Beijing University of Posts and Telecommunications (BUPT), Beijing 100876, China; cbb0528@bupt.edu.cn (B.C.); lqwangwang@bupt.edu.cn (L.W.); zhangzhiguo@bupt.edu.cn (Z.Z.); 2 State Key Laboratory of Information Photonics and Optical Communications, Beijing University of Posts and Telecommunications (BUPT), Beijing 100876, China; 3 School of Physics and Optoelectronic Engineering, Nanjing University of Information Science and Technology, Nanjing 211544, China; 003101@nuist.edu.cn

**Keywords:** hybrid FSO/RF, deep reinforcement learning (DRL), proximal policy optimization (PPO), trajectory optimization

## Abstract

Space–air–ground systems employing free-space optical (FSO) communication leverage high-altitude platform stations (HAPS) to deliver seamless and ubiquitous connectivity. Although FSO links offer high capacity, they are highly susceptible to cloud extinction, which severely degrades link availability. Hybrid FSO/radio-frequency (RF) transmission and cloud-aware HAPS trajectory optimization can enhance resilience. However, the conventional cloud-aware hybrid FSO/RF transmission system based on hard-switching (HS) between the FSO and RF links leads to frequent link transitions and unstable throughput. To address these challenges, we propose a joint optimization framework that integrates soft-switch between FSO and RF links with deep reinforcement learning (DRL) for HAPS trajectory optimization. Soft-switching based on rateless codes (RCs) enables simultaneous transmission over both links, where the receiver accumulates packets until successful decoding with a single feedback. The feedback frequency of RC is sparse, which avoids feedback storms but also poses challenges to HAPS trajectory optimization. The DRL agent proactively optimizes HAPS trajectories to avoid cloud cover and maintain link availability. To address the sparse feedback of RCs for DRL training, a reward-shaped proximal policy optimization (PPO)-based agent is developed to jointly optimize throughput and trajectory smoothness. Simulations using realistic ERA5 data show that RC-PPO achieves higher throughput and smoother trajectories compared to the HS-PPO baseline.

## 1. Introduction

The explosive growth of global data traffic has revealed the limitations of terrestrial networks in achieving ubiquitous connectivity [1]. Space–air–ground integrated networks (SAGINs) with free-space optical communication (FSO), using high-altitude platform stations (HAPS) to relay communications between satellites and ground stations, have emerged as a promising solution [2,3]. FSO links offer ultra-high bandwidth but suffer from low reliability due to atmospheric conditions such as clouds and atmospheric turbulence (AT) [4,5]. Hybrid FSO/RF transmission systems leverage RF backup to enhance reliability [6,7,8]. Such hybrid systems have been deployed in dual-hop relay links [9] and SAGIN scenarios to improve coverage and capacity [10]. Conventional hybrid FSO/RF systems employ hard-switching (HS) strategies that select links based on instantaneous SNR values [11], suffering from frequent transitions and unstable throughput. FSO/RF systems based on rateless code (RC), such as Raptor code [12], enable soft-switching by transmitting distinct encoded packets simultaneously over both the FSO and RF channels [13], thus eliminating switching overhead and improving reliability against burst disruptions. To avoid cloud cover, HAPS trajectory optimization is also an effective method to enhance link stability and throughput.

Deep reinforcement learning (DRL) offers a powerful framework for adaptive HAPS trajectory optimization in dynamic atmospheric environments [14]. For example, ref. [15] integrates 5G low-latency connectivity with a Deep Q-Network (DQN) for 3D trajectory optimization. Ref. [16] employs the deep deterministic policy gradient (DDPG) algorithm for coordinating HAPS coverage. However, these studies foucus on RF-only aerial communication networks. When extending DRL to hybrid FSO/RF systems, ref. [17] applies the A2C algorithm to optimize the HAPS trajectory in the HS FSO/RF transmission system, which relies on dense, per-step reward signals. This dense-reward strategy is fundamentally incompatible with the RC-based soft-switching FSO/RF system. For RC, a meaningful reward is available only upon successful block decoding, which introduces a critical challenge of reward sparsity. This sparse feedback (reward) severely complicates temporal credit assignment and hinders efficient exploration [18]. Ref. [19] successfully used Proximal Policy Optimization (PPO) with reward shaping to compensate for such reward sparsity when guiding UAVs to move to destinations. To the best of our knowledge, no existing work has investigated HAPS trajectory planning in an RC-based soft-switching hybrid FSO/RF system under stochastic cloud dynamics.

This paper proposes a joint optimization framework that integrates rateless-coded physical-layer transmission with PPO-based trajectory learning in a hybrid FSO/RF transmission system. Key contributions are as follows:

- The cloud-aware HAPS trajectory optimization problem in soft-switching hybrid FSO/RF systems is formulated and solved by a PPO-based DRL approach, under the stochastic moving occluding cloud (SMOC) model derived from the ERA5 dataset.
- A potential-based reward-shaping mechanism within the PPO framework is developed to mitigate sparse decoding feedback of RCs, delivering faster convergence and superior performance over threshold-based HS-PPO schemes.

The remainder of this paper is organized as follows. Section 2 presents the scheme of the trajectory-optimized HAPS–ground station (GS) hybrid FSO/RF link, FSO/RF channel models, and the throughput of HS and RC-based hybrid FSO/RF systems. Section 3 details the proposed RC-PPO algorithm and the HS-PPO algorithm as a reference and presents the corresponding Markov Decision Process (MDP) formulation and reward shaping strategy. Section 4 provides simulation results. Section 5 gives the conclusion.

## 2. System Model

This section presents the mathematical framework for the HAPS-assisted hybrid FSO/RF communication system. Firstly, a simplified three-tier SAGIN architecture is introduced. Subsequently, the channel model of FSO and RF links are presented. At last, the throughputs for hybrid FSO/RF systems with HS and RC are elaborated.

### 2.1. Space–Air–Ground Architecture

**Figure 1:**
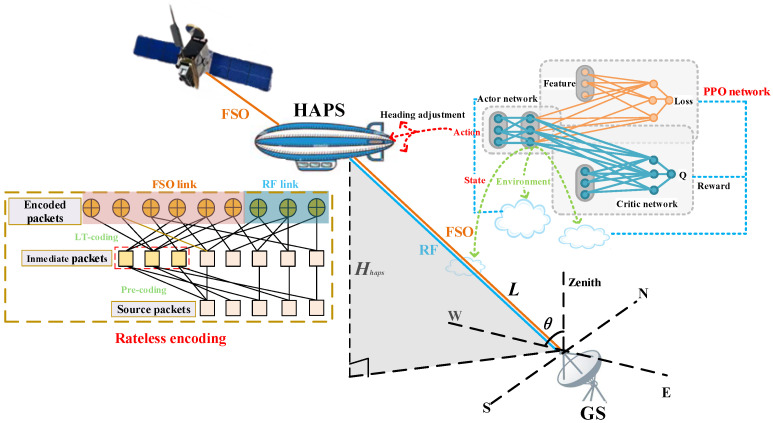
Soft-switching hybrid FSO/RF SAG system.

As illustrated in Figure 1, we consider a simplified three-tier Space–Air–Ground (SAG) system, which constitutes a fundamental building block of standard SAGINs. The LEO satellite operates at an orbital altitude of approximately 600 km and serves as the primary data source. The HAPS, positioned at $H_{haps}=20$ km in the stratosphere, functions as an aerial relay node to bridge the satellite-to-ground communication gap. The satellite-to-HAPS link employs FSO communication to exploit its high bandwidth capacity, while the HAPS-to-GS link utilizes hybrid FSO/RF transmission to provide complementary link diversity.

A discrete-time model is adopted with time step index t∈{1,2,…,T}. The position of HAPS at time *t* is denoted as $p_{h}\left[t\right]=\left(x\left[t\right],y\left[t\right],H_{haps}\right)$, where (x[t],y[t]) represents the horizontal coordinates and $H_{haps}$ (the altitude of HAPS) remains constant. The GS is located at $p_{g}=\left(0,0,H_{gs}\right)$ with $H_{gs}=0$. The instantaneous slant distance between HAPS and GS is given by:

(1) $$L\left[t\right]=∥p_{h}\left[t\right]-p_{g}∥=\sqrt{x{\left[t\right]}^{2}+y{\left[t\right]}^{2}+H_{haps}^{2}}.$$

And the elevation angle (Figure 1) is $\theta \left[t\right]=\arctan\left(H_{haps}/\sqrt{x{\left[t\right]}^{2}+y{\left[t\right]}^{2}}\right)$.

### 2.2. Channel Model

#### 2.2.1. FSO Channel Model

The composite channel gain of the downlink FSO channel between HAPS and GS is expressed as:

(2) $$H_{\mathrm{FSO}}\left[t\right]=h_{g}\left[t\right]\cdot h_{a}\left[t\right]\cdot h_{l}\left[t\right],$$

where $h_{g}\left[t\right]$ is the geometric loss due to the divergence of the beam on the slant path distance L[t] [20], $h_{a}\left[t\right]$ follows the Gamma-Gamma distribution characterizing AT-induced fading [21], and $h_{l}\left[t\right]$ represents the loss of cloud-induced attenuation. Among these factors, $h_{l}\left[t\right]$ is critical for optimizing the HAPS trajectory due to its spatial heterogeneity. The cloud-induced attenuation follows the Beer–Lambert law [22]:

(3) $$h_{l}\left[t\right]=\exp\left(-\sigma L\left[t\right]\right).$$

σ (km^−1^) is the attenuation coefficient [23], determined based on the visibility (*V*) (in km) and the wavelength (in nm) as

(4) $$\sigma =\frac{3.91}{V}{\left(\frac{\lambda }{550}\right)}^{-q}.$$

Here, λ is the optical wavelength, *V* is the visibility, and *q* is the size-distribution parameter of the scattering particles depending on *V*. The expression of *V* is given by:

(5) $$V=-\frac{\ln(0.002)}{\beta_{f}\left[t\right]},$$

where $\beta^{f}\left[t\right]$ is the cloud-induced attenuation of the FSO link obtained by integration over the slant path:

(6) $$\beta^{f}\left[t\right]=\frac{1}{\sin(\theta [t])}\int_{h_{0}}^{h_{\mathrm{top}}}\beta_{\mathrm{ext}}^{f}\left(h\right)\;dh.$$

Here, θ[t] is the elevation angle and $h_{0}$ and $h_{\mathrm{top}}$ are the cloud base and top altitudes. The extinction coefficient $\beta_{\mathrm{ext}}^{f}\left(h\right)$ for the FSO link is given by [24]:

(7) $$\beta_{\mathrm{ext}}^{f}\left(h\right)=\frac{6.51\times 10^{3}\cdot c\left(h\right)}{\rho_{w}\cdot r_{e}},$$

where $\rho_{w}=1$ g/cm^3^ is the water density, $r_{e}$ (μm) is the particle effective radius [24], and c(h) is the vertical profile of liquid water content (LWC) in g/m^3^. The three-dimensional (3D) distribution of LWC is modeled as a spatially correlated stochastic field, as detailed in Section 4.1.

The FSO link employs intensity modulation with direct detection (IM/DD) using on–off keying (OOK). The received optical power is $P_{\mathrm{rx}}^{f}\left[t\right]=P_{\mathrm{tx}}^{f}G_{\mathrm{tx}}^{f}G_{\mathrm{rx}}^{f}H_{\mathrm{FSO}}\left[t\right]$, where $P_{\mathrm{tx}}^{f}$ denotes the transmit optical power and $G_{\mathrm{tx}}^{f}$ and $G_{\mathrm{rx}}^{f}$, respectively, denote the transmitter and receiver telescope gains. The instantaneous electrical SNR is given by:

(8) $$\gamma_{\mathrm{FSO}}\left[t\right]=\frac{{\left(RP_{\mathrm{rx}}^{f}\left[t\right]\right)}^{2}}{N_{0}^{f}B_{f}},$$

where *R* (A/W) is the photodetector responsivity, $N_{0}^{f}$ (A^2^/Hz) is the noise power spectral density, and $B_{f}$ (Hz) is the receiver bandwidth of the FSO link.

#### 2.2.2. RF Channel Model

The RF channel provides complementary connectivity with enhanced reliability under cloud-obscured conditions. The RF channel gain incorporates free-space path loss (FSPL) and cloud-induced attenuation. The expression of FSPL is given by:

(9) $${\mathrm{FSPL}}_{r}\left[t\right]={\left(\frac{4\pi L\left[t\right]f_{c}}{c_{0}}\right)}^{2},$$

with RF carrier frequency $f_{c}$ and speed of light $c_{0}$. The cloud attenuation coefficient $\beta^{r}\left[t\right]$ (km^−1^) is computed similarly to (6) for FSO links by path-integrating the specific attenuation coefficient. The specific RF attenuation coefficient $\beta_{\mathrm{ext}}^{r}\left(h\right)$ (km^−1^) is derived from Mie scattering theory and modeled as [25]:

(10) $$\beta_{\mathrm{ext}}^{r}\left(h\right)=k_{\mathrm{ext}}^{r}\cdot c\left(h\right),$$

where $k_{\mathrm{ext}}^{r}$ (km^−1^/(g·m^−3^)) is the mass extinction coefficient at RF frequency $f_{c}$.

Similar to (8), the instantaneous SNR of RF link with QPSK modulation is given by:

(11) $$\gamma_{\mathrm{RF}}\left[t\right]=\frac{P_{\mathrm{tx}}^{r}G_{\mathrm{tx}}^{r}G_{\mathrm{rx}}^{r}H_{\mathrm{RF}}\left[t\right]}{N_{0}^{r}B_{r}},$$

where $P_{\mathrm{tx}}^{r}$ is the transmitting power, $G_{\mathrm{tx}}^{r}$ and $G_{\mathrm{rx}}^{r}$ are the transmitting and receiving antenna gains, $N_{0}^{r}$ (W/Hz) is the spectral density of RF noise power, and $B_{r}$ (Hz) is the receiver bandwidth of the RF link.

### 2.3. Hybrid FSO/RF Systems

This section describes the throughput of hybrid FSO/RF communication systems based on HS and RC. These two schemes differ fundamentally in how they exploit link diversity, which affects trajectory optimization.

#### 2.3.1. Hard Switching

The HS scheme selects either the FSO or RF link at each time step based on instantaneous channel quality. The instantaneous throughput is given by:

(12) $$T_{\mathrm{HS}}\left[t\right]=\left\{\begin{array}{cc} R_{\mathrm{FSO}}\;P_{\mathrm{suc}}^{\mathrm{FSO}}\left[t\right], & \mathrm{if}\;\gamma_{\mathrm{FSO}}\left[t\right]\geq \gamma_{\mathrm{th}},\\ R_{\mathrm{RF}}\;P_{\mathrm{suc}}^{\mathrm{RF}}\left[t\right], & \mathrm{if}\;\gamma_{\mathrm{FSO}}\left[t\right]<\gamma_{\mathrm{th}}, \end{array}\right.$$

where $\gamma_{\mathrm{th}}$ is the switching threshold, $R_{\mathrm{FSO}}$ and $R_{\mathrm{RF}}$ are the transmitted data rates, $P_{\mathrm{suc}}^{\mathrm{FSO}}\left[t\right]$ and $P_{\mathrm{suc}}^{\mathrm{RF}}\left[t\right]$ are the per-packet success reception probabilities accounting for forward error correction (FEC). The optimal threshold value maximizes the expected throughput:

(13) $$\gamma_{\mathrm{th}}^{★}=\arg\max_{\gamma_{\mathrm{th}}}E\left[T_{\mathrm{HS}}\left[t\right]\right].$$

For the hybrid FSO/RF system, the bit error rate (BER) for the FSO link with OOK modulation and the RF link with QPSK modulation depend on their respective signal-to-noise ratios (SNRs) at the receiver. The BER for each link can be uniformly expressed as:

(14) $${\mathrm{BER}}_{i}\left[t\right]=\frac{1}{2}\mathrm{erfc}\left(\sqrt{\kappa_{i}\cdot \gamma_{i}\left[t\right]}\right),$$

where i∈{f,r} denotes the FSO or RF link, $\gamma_{i}\left[t\right]$ is the instantaneous SNR, and $\kappa_{i}$ is the modulation-dependent efficiency factor, with $\kappa_{f}=0.5$ for OOK and $\kappa_{r}=1$ for QPSK. For a packet of $L_{p}$ bits in length, assuming FEC capable of correcting up to $t_{\mathrm{corr}}$ bit errors, the packet success probability is computed via the binomial sum:

(15) Psuc[t]=∑i=0tcorrLpiBER[t]i1−BER[t]Lp−i.

#### 2.3.2. Rateless Coding

RCs enable the generation of an arbitrary number of encoded packets until the receiver accumulates sufficient packets to decode the original source block [13]. Unlike traditional channel codes with fixed code rates, RCs automatically adjust the number of encoded packets required to be sent for successful decoding based on channel conditions. Raptor code, which represents the state of the art in RCs, consists of a high-code-rate precode (typically Low-Density Parity-Check (LDPC) code [26]) followed by a Luby Transform (LT) [27] outer code and is utilized in this paper.

Let $s\in {\mathrm{GF}}_{2}^{N_{s}}$ denote the source symbol vector containing $N_{s}$ information symbols. The precode generates an intermediate symbol vector (Figure 1):

(16) $$c=G_{\mathrm{pre}}s,$$

where $G_{\mathrm{pre}}$ is the precode generator matrix producing $N_{c}$ intermediate symbols. The LT encoder then produces output symbols via:

(17) $$x_{i}=\overset{N_{c}}{\underset{j=1}{⨁}}G_{\mathrm{LT}}\left(i,j\right)\;c_{j},$$

where $G_{\mathrm{LT}}\left(i,\cdot \right)$ is a random sparse row sampled according to a robust soliton degree distribution and ⊕ denotes XOR over ${\mathrm{GF}}_{2}$. Each output symbol $x_{i}$ is formed by XORing a small subset of intermediate symbols, with the subset size and selection determined by the degree distribution.

In the RC scheme, both FSO and RF links transmit different encoded symbols simultaneously. Crucially, any correctly received symbol contributes to decoding, regardless of which link delivered it. This superposition property eliminates the need for link selection decisions and enables full utilization of both links under all channel conditions. The receiver can successfully decode the source data block once it accumulates approximately $N_{s}\left(1+\varepsilon_{c}\right)$ distinct encoded symbols, where $\varepsilon_{c}$ is the average overhead required by the belief-propagation (BP) decoder. This overhead is typically $\varepsilon_{c}\approx 0.05$–0.1 for well-designed Raptor codes [13]. The impact of dynamic SNR fluctuations on the hybrid link is captured through the packet success probabilities $P_{suc}^{FSO}$ and $P_{suc}^{RF}$ in Equation (15), which determine the rate of correctly received symbols. Therefore, the expected effective throughput for RC is given by:

(18) $$E\left[T_{\mathrm{RC}}\right]=\frac{R_{\mathrm{FSO}}\;P_{\mathrm{suc}}^{\mathrm{FSO}}+R_{\mathrm{RF}}\;P_{\mathrm{suc}}^{\mathrm{RF}}}{1+\varepsilon_{c}}.$$

## 3. Trajectory Optimization

This section presents the trajectory optimization framework for hybrid FSO/RF communication schemes. Firstly, the PPO algorithm and MDP formulation are introduced as the foundation of DRL. Subsequently, the trajectory optimization strategies for the HS and RC schemes using PPO-based DRL are elaborated.

### 3.1. Proximal Policy Optimization

PPO is an on-policy actor-critic algorithm that uses a clipped surrogate objective to constrain policy updates, thereby preventing large deviations from the current policy and ensuring stable training. The objective of PPO, which is maximized at each iteration, is given by:

(19) $$L_{t}^{\mathrm{PPO}}\left(\theta \right)={\hat{E}}_{t}\left[L_{t}^{\mathrm{CLIP}}\left(\theta \right)-c_{1}L_{t}^{\mathrm{VF}}\left(\theta \right)+c_{2}H\left[\pi_{\theta }\right]\left(s_{t}\right)\right],$$

where ${\hat{E}}_{t}\left[\cdot \right]$ denotes the empirical average over a finite batch of samples, $H\left[\pi_{\theta }\right]\left(s_{t}\right)$ denotes an entropy bonus, $L_{t}^{\mathrm{VF}}\left(\theta \right)$ is the value function loss, and $c_{1},c_{2}>0$ are weighting coefficients.

The clipped surrogate objective $L_{t}^{\mathrm{CLIP}}\left(\theta \right)$ prevents excessively large policy updates by constraining the probability ratio between the current and old policies, given by:

(20) $$L_{t}^{\mathrm{CLIP}}\left(\theta \right)={\hat{E}}_{t}\;\left[\min\;\left(\mu_{t}\left(\theta \right){\hat{A}}_{t},\;\mathrm{clip}\;\left(\mu_{t}\left(\theta \right),1-\epsilon_{\mathrm{clip}},1+\epsilon_{\mathrm{clip}}\right){\hat{A}}_{t}\right)\right],$$

where the function clip $\left(\mu_{t}\left(\theta \right),1-\varepsilon_{\mathrm{clip}},1+\varepsilon_{\mathrm{clip}}\right)$ acts as a clipping operation that constrains the probability ratio $\mu_{t}\left(\theta \right)$ to the interval $\left[1-\varepsilon_{\mathrm{clip}},\;1+\varepsilon_{\mathrm{clip}}\right]$. $\epsilon_{\mathrm{clip}}$ (typically 0.1–0.3) is a hyperparameter. The probability ratio $\mu_{t}\left(\theta \right)$ at time step *t* is given by:

(21) $$\mu_{t}\left(\theta \right)=\frac{\pi_{\theta }\left(a_{t}|s_{t}\right)}{\pi_{\theta_{\mathrm{old}}}\left(a_{t}|s_{t}\right)}.$$

Moreover, the advantage estimator (${\hat{A}}_{t}$) is formulated as a discounted sum of future temporal-difference (TD) residuals ($\delta_{t}$), given by:

(22) $${\hat{A}}_{t}=\sum_{l=0}^{T-t}\gamma^{l}\delta_{t+l},$$

where γ∈(0,1) is the discount factor, and the TD residual is defined as:

(23) $$\delta_{t}=r_{t}+\gamma V\left(s_{t+1}\right)-V\left(s_{t}\right).$$

Here, $r_{t}$ is the immediate reward received from the environment, and $V(s_{t})$ is the state value, which can be used to evaluate the state $s_{t}$ under the current policy.

The value function loss ($L_{t}^{\mathrm{VF}}\left(\theta \right)$) minimizes the mean squared error (MSE) between predicted state values and empirical returns, given by:

(24) $$L_{t}^{\mathrm{VF}}\left(\theta \right)={\hat{E}}_{t}\;\left[{\left(V_{\theta }\left(s_{t}\right)-R_{t}\right)}^{2}\right],$$

where $R_{t}=\sum_{l=0}^{T-t}\gamma^{l}r_{t+l}$ is the discounted sum of future rewards. The entropy term ($H[\pi_{\theta }]$) encourages exploration by preventing premature convergence to deterministic policies, given by

(25) $$H\left[\pi_{\theta }\right]\left(s_{t}\right)=-E_{a∼\pi_{\theta }\left(\cdot |s_{t}\right)}\left[\log\pi_{\theta }\left(a|s_{t}\right)\right].$$

Network parameters are updated after collecting a trajectory of length *T*, using the collected batch to perform multiple epochs of minibatch gradient descent on the PPO objective. The pseudocode of the algorithm is shown in Algorithm 1.
**Algorithm 1** PPO for HAPS Trajectory Optimization
**Require:** Policy and value network $\pi_{\theta },V_{\theta }$, hyperparameters $\gamma ,\epsilon_{\mathrm{clip}},c_{1},c_{2},K$
1:**for** each training iteration **do**2:      Collect trajectory $D={\left\{\left(s_{t},a_{t},r_{t},s_{t+1}\right)\right\}}_{t=0}^{T}$ using $\pi_{\theta }$3:      Compute returns $R_{t}=\sum_{l=0}^{T-t}\gamma^{l}r_{t+l}$4:      Compute TD residuals $\delta_{t}=r_{t}+\gamma V_{\theta }\left(s_{t+1}\right)-V_{\theta }\left(s_{t}\right)$5:      Compute advantages ${\hat{A}}_{t}=\sum_{l=0}^{T-t}\gamma^{l}\delta_{t+l}$6:      Normalize advantages: ${\hat{A}}_{t}\leftarrow \left({\hat{A}}_{t}-\mu \right)/\sigma$7:      Store old policy probabilities $\pi_{\theta_{\mathrm{old}}}\left(a_{t}|s_{t}\right)$8:      **for** epoch k=1,…,K **do**9:            **for** mini-batch B⊂D **do**10:                  Compute $L_{t}^{\mathrm{PPO}}\left(\theta \right)=L_{t}^{\mathrm{CLIP}}\left(\theta \right)-c_{1}L_{t}^{\mathrm{VF}}\left(\theta \right)+c_{2}H\left[\pi_{\theta }\right]\left(s_{t}\right)$11:                  Update θ via gradient ascent on $L_{t}^{\mathrm{PPO}}\left(\theta \right)$12:            **end for**13:      **end for**14:**end for**15:**return** Optimized policy $\pi_{\theta^{*}}$

### 3.2. Trajectory Optimization with PPO-Based DRL

The HAPS trajectory optimization problem is formalized as a finite-horizon MDP defined by the tuple M=(S,A,P,R,γ), where S is the state space, A is the action space, *P* is the transition probability, and *R* is the reward function. The control objective for both RC and HS schemes is to find an optimal policy maximizing the expected discounted return:

(26) $$\pi^{*}=\arg\max_{\pi_{\theta }}E_{s_{0}∼\mu ,a_{t}∼\pi_{\theta }\left(\cdot |s_{t}\right),s_{t+1}∼P\left(\cdot |s_{t},a_{t}\right)}\left[\sum_{t=0}^{T}\gamma^{t}r_{t}\right].$$

The state space, action space, and reward function differ significantly between the RC and HS schemes due to their distinct communication paradigms.

#### 3.2.1. RC-PPO

The state space should contain all information needed for the RC-PPO agent to make decisions and preserve the Markovian property of the environment, given by:

(27) $$s_{t}^{\mathrm{RC}}=\left(x_{t},y_{t},\psi_{t},\tau_{t},\rho_{t}\right)\in S^{\mathrm{RC}},$$

where $\left(x_{t},y_{t}\right)\in R^{2}$ denote the current coordinates of the HAPS, $\psi_{t}\in \left[0,2\pi \right)$ is the heading angle measured clockwise from north, $\tau_{t}=T-t$ is the number of steps remaining, and $\rho_{t}=\left[\rho \left(p_{t,1}\right),\ldots ,\rho \left(p_{t,N_{p}}\right)\right]\in {\left[0,1\right]}^{N_{p}}$ contains cloud extinction coefficients sampled at $N_{p}$ probe points $\left\{p_{t,i}\right\}$ along prospective directions.

Since RC allows both links to transmit simultaneously without switching overhead, the action space contains only navigation decisions:

(28) $$a_{t}^{\mathrm{RC}}=\Delta \psi_{t}\in A^{\mathrm{RC}},$$

where $\Delta \psi_{t}\in \left[-\psi_{\max},+\psi_{\max}\right]$ is the discrete heading change with $\psi_{\max}=30^{\circ }$ representing the maximum single-step turn angle.

State transitions decompose into deterministic kinematic evolution and stochastic cloud field dynamics. For simplicity, the speed of the HAPS is assumed constant of $v_{0}$, and the effects of wind (atmospheric currents) are not considered in the kinematic model. The HAPS position evolves according to:

(29) $$\begin{array}{cc} x_{t+1} & =x_{t}+v_{0}\Delta t\cos\left(\psi_{t}+\Delta \psi_{t}\right),\\ y_{t+1} & =y_{t}+v_{0}\Delta t\sin\left(\psi_{t}+\Delta \psi_{t}\right),\\ \psi_{t+1} & =(\psi_{t}+\Delta \psi_{t})\;\mathrm{mod}\;2\pi . \end{array}$$

The decoding progress $\eta_{t}\in \left[0,1\right]$, defined as the fraction of successfully received packets to the total required, evolves according to:

(30) $$\eta_{t+1}=\min\left(1,\eta_{t}+\frac{\Lambda_{t}^{\mathrm{RC}}\Delta t}{N_{s}\left(1+\varepsilon_{c}\right)}\right),$$

where $N_{s}$ is the total number of source packets, $\varepsilon_{c}$ is the redundancy overhead in the RCs, and $\Lambda_{t}^{\mathrm{RC}}$ is the aggregate packet-arrival rate from both links. Noted, in real-world deployment, the receiver provides feedback only upon successful decoding of complete data blocks, making $\eta_{t}$ unavailable for real-time decision-making by the HAPS. The aggregate rate:

(31) $$\Lambda_{t}^{\mathrm{RC}}=\sum_{i=1}^{N_{p}}w_{i}\left(R_{\mathrm{FSO}}\cdot P_{suc}^{\mathrm{FSO}}\left(\rho \left(p_{t,i}\right)\right)+R_{\mathrm{RF}}\cdot P_{suc}^{\mathrm{RF}}\left(\rho \left(p_{t,i}\right)\right)\right),$$

where $R_{\mathrm{FSO}}$ and $R_{\mathrm{RF}}$ are the transmitted symbol rates of the FSO and RF links, $P_{suc}^{\mathrm{FSO}}\left(\rho \right)$ and $P_{suc}^{\mathrm{RF}}\left(\rho \right)$ denote the probability of successful symbol reception as a function of extinction loss of cloud, and $w_{i}=\exp\left(-d_{i}/d_{0}\right)$ are distance-dependent weight factors where $d_{i}$ is the distance from HAPS to the *i*th probe position and $d_{0}$ is a normalization constant, thereby assigning higher influence to nearer probes. At each time step, $N_{p}$ probe points ${\left\{p_{t,i}\right\}}_{i=1}^{N_{p}}$ are placed along candidate directions at varying distances from the current HAPS position to estimate future channel conditions.

In order to maximize the average capacity over the allowed *T* time steps, the reward function balances multiple objectives and is defined as:

(32) $$r_{t}^{\mathrm{RC}}=\alpha \Lambda_{t}^{\mathrm{RC}}+\beta \Delta \eta_{t}-\kappa_{\psi }\left|\Delta \psi_{t}\right|-\kappa_{d}{\left(\frac{d_{t}}{d_{0}}\right)}^{2}+qR_{\mathrm{suc}},$$

where $\Lambda_{t}^{\mathrm{RC}}$ encourages high instantaneous throughput, $\Delta \eta_{t}=\eta_{t+1}-\eta_{t}$ provides dense feedback on decoding progress through potential-based reward shaping, $|\Delta \psi_{t}|$ discourages sharp heading changes to maintain flight stability, and ${\left(d_{t}/d_{0}\right)}^{2}$ with $d_{t}=\sqrt{x_{t}^{2}+y_{t}^{2}}$ penalizes excessive distance from the GS to accelerate policy convergence speed and improve link quality. α, β, $\kappa_{\psi }$, $\kappa_{d}$, and *q* are weighting factors to balance the physical and task-driven scales of their corresponding terms.

The terminal reward $R_{\mathrm{suc}}$ indicates mission completion:

(33) $$R_{\mathrm{suc}}=\left\{\begin{array}{cc} 1, & \mathrm{if}\;\eta_{t}=1\;\mathrm{and}\;t\leq T,\\ 0, & \mathrm{otherwise}. \end{array}\right.$$

An episode terminates when either the mission succeeds ($R_{\mathrm{suc}}=1$) or timeout occurs ($\tau_{t}=0$).

The inclusion of $\Delta \eta_{t}$ in the reward function addresses the sparse feedback challenge inherent in RC. According to the potential-based shaping theorem [19], our shaping term $\beta \Delta \eta_{t}$ corresponds to the potential function $\Phi \left(s\right)=\beta \eta_{t}$, which preserves the optimal policy while providing dense training signals.

Importantly, although $\eta_{t}$ is used during training to accelerate policy learning, the trained policy $\pi_{\theta }\left(a_{t}^{\mathrm{RC}}|s_{t}^{\mathrm{RC}}\right)$ depends only on the observable state $s_{t}^{\mathrm{RC}}=\left(x_{t},y_{t},\psi_{t},\tau_{t},\rho_{t}\right)$ and requires no real-time decoding feedback at deployment.

#### 3.2.2. HS-PPO

For HS-PPO, the state space omits decoding progress but includes the currently selected link represented by the binary indicator $H_{t}$:

(34) $$s_{t}^{\mathrm{HS}}=\left(x_{t},y_{t},\psi_{t},\tau_{t},H_{t},\rho_{t}\right)\in S^{\mathrm{HS}},$$

where $H_{t}\in \left\{\mathrm{FSO},\mathrm{RF}\right\}$ denotes the currently active link. The action space includes both navigation and link selection decisions:

(35) $$a_{t}^{\mathrm{HS}}=\left(\Delta \psi_{t},h_{t}\right)\in A^{\mathrm{HS}},$$

where $h_{t}\in \left\{0,1\right\}$ denotes the currently active link (0 for FSO, 1 for RF). The instantaneous reward is defined as:

(36) $$r_{t}^{\mathrm{HS}}=\alpha \Lambda_{t}^{\mathrm{HS}}-\kappa_{\psi }\left|\Delta \psi_{t}\right|-\xi I\left[H_{t}\neq H_{t-1}\right]-\kappa_{d}{\left(\frac{d_{t}}{d_{0}}\right)}^{2}+qR_{\mathrm{suc}},$$

where ξ>0 penalizes frequent link switch, and $\Lambda_{t}^{\mathrm{HS}}$ is the data rate from the active link:

(37) $$\Lambda_{t}^{\mathrm{HS}}=\left\{\begin{array}{cc} R_{\mathrm{FSO}}\cdot P_{suc}^{\mathrm{FSO}}\left(\rho \left(p_{t}\right)\right), & \mathrm{if}\;H_{t}=\mathrm{FSO},\\ R_{\mathrm{RF}}\cdot P_{suc}^{\mathrm{RF}}\left(\rho \left(p_{t}\right)\right), & \mathrm{if}\;H_{t}=\mathrm{RF}. \end{array}\right.$$

The terminal reward $R_{\mathrm{suc}}$ for HS-PPO is defined similar to that for RC-PPO, with episodes terminating upon mission completion or timeout.

## 4. Simulation and Results

This section presents the cloud field generation, training setup, and evaluation of the proposed HS-PPO and RC-PPO agents. Key parameters are summarized in Table 1.

### 4.1. Cloud Field Generation

The 3D LWC field is generated using the SMOC model [28], leveraging statistics of cloud cover and average integrated liquid water content (ILWC) from the ERA5. A log-normal distribution of the content of liquid cloud water (CLWC) over the given site is obtained based on the extracted information from the dataset. Spatially correlated 3D LWC fields are then synthesized by generating random Gaussian fields based on empirical spatial correlations from ERA5, ensuring realistic cloud structures. Finally, an analytical vertical profile c(h) is applied to modulate the altitude-dependent LWC distribution within each cloud column:

(38) $$c\left(h\right)=\left\{\begin{array}{cc} \frac{W}{b_{c}^{a_{c}}\Gamma \left(a_{c}\right)}{\left(h-h_{0}\right)}^{a_{c}-1}e^{-\left(h-h_{0}\right)/b_{c}}, & h\geq h_{0}\\ 0, & h<h_{0} \end{array}\right.$$

where *W* is the columnar ILWC (in kg/m^2^), $h_{0}$ is the cloud base altitude, Γ(·) denotes the gamma function, and $a_{c}$, $b_{c}$ determine the shape of the clouds’ vertical profile, given by:

(39) $$\begin{array}{cc} a_{c} & =4.27\exp[-4.93(C+0.06)]+54.12\exp[-61.25(C+0.06)]+1.71,\\ b_{c} & =3.17a_{c}^{-3.04}+0.074, \end{array}$$

where *C* is the CLWC. The resulting 3D LWC field is used to compute the specific extinction coefficients of the cloud for the FSO and RF channels according to (7) and (10), respectively.

### 4.2. PPO Training Configuration

Both RC-PPO and HS-PPO agents employ identical neural network architectures, consisting of a three-layer MLP feature extractor with hidden dimensions [128, 64, 32] and ReLU activations. The feature extractor feeds into separate policy and value heads. The policy head was initialized using orthogonal initialization with a gain of 0.01 to reduce initial entropy. Each cycle collects 200 episodes to form a batch. This batch is reused for 10 update epochs with a mini-batch size of 64. Evaluation is performed with 200 deterministic test episodes employing the static cloud fields generated in Section 4.1. PPO training hyperparameters are detailed in Table 2.

Moreover, for the weighting parameter settings of the reward function (Equations (32) and (35)), an iterative behavior-driven tuning methodology is employed. Specifically, for the RC-PPO agent, α is fixed as the baseline to reflect the primary objective of maximizing throughput; β is selected via grid search to balance the decoding progress signal and throughput exploration; $\kappa_{\psi }$ and $\kappa_{d}$ serve as regularization terms to encourage smooth trajectories and movement toward the ground station, respectively. *q* is set to a large scalar to clearly signify task completion. The specific parameter values are summarized in Table 3.

### 4.3. Results and Discussion

**Figure 2:**
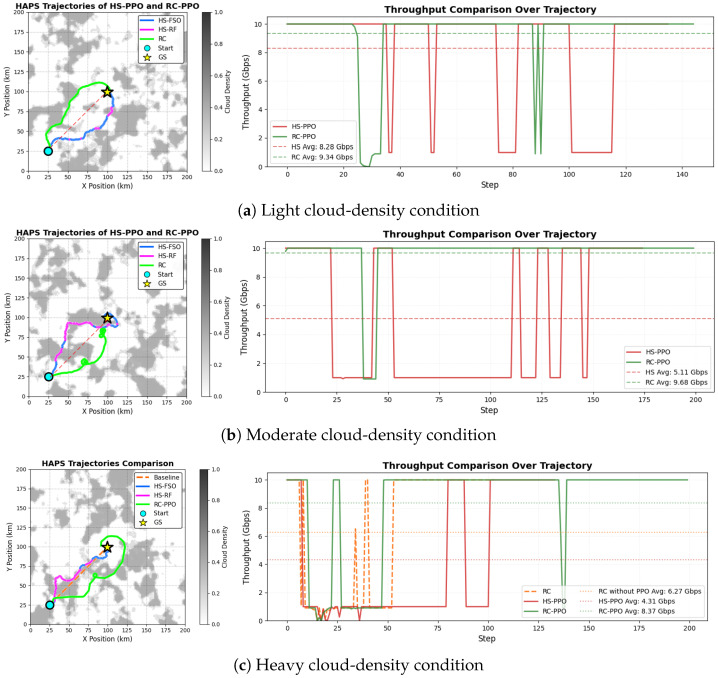
HAPS trajectories and instantaneous throughput for HS-PPO and RC-PPO scheme under (**a**) light, (**b**) moderate, and (**c**) heavy cloud-density conditions.

Figure 2 shows representative HAPS trajectories and their corresponding instantaneous throughput achieved by the HS-PPO and RC-PPO agents under varied cloud conditions. To systematically assess the performance of both schemes, 100 random cloud distributions were first generated using the SMOC model. For each cloud distribution, the ILWC along the slant path was averaged over the starting point to the GS (indicated by the red dashed line in Figure 2). The mean values of ILWC were then ranked across 100 cloud distribution cases to approximately characterize the difficulty of trajectory optimization. For each typical HAPS mission duration (approximately 0.8–1.3 h for a 100 km flight at 75–120 km/h [29]), the cloud field can be regarded as slowly varying (with a temporal decorrelation scale of 15–29 h [30]). Hence, the use of a static cloud map in each episode is justified.

Representative cloud scenarios were selected at the 25th, 50th, and 75th among 100 cloud distributions of this ranked distribution, corresponding to light, moderate, and heavy cloud-density distributions. The horizontal position of the starting point is uniformly fixed at (25 km, 25 km), and the position of the GS is set at (100 km, 100 km).

The overall objective of HAPS trajectory optimization is to approach the GS while actively avoiding dense cloud regions, thereby minimizing signal attenuation and maximizing both throughput and communication coverage of the HAPS. Under light cloud-density conditions (Figure 2a), both HS-PPO and RC-PPO agents tend to advance directly toward the GS, achieving favorable throughput performance due to minimal cloud-induced extinction. As cloud density increases (Figure 2b,c), HS-PPO exhibits substantial throughput degradation attributed to severe FSO link attenuation in dense cloud regions, necessitating RF channel backup. In contrast, RC-PPO achieves higher effective hybrid throughput by dynamically leveraging transient FSO transmission windows while maintaining a reliable RF backup link. This strategy enables RC-PPO to reduce signal interruption durations and feedback latency compared to HS-PPO.

In terms of quantified throughput, under light cloud, the average throughput of RC-PPO is 9.34 Gbps, which is improved by 12.8% compared with the throughput of 8.28 Gbps for HS-PPO. As cloud density increases to a moderate level, RC-PPO reaches 9.68 Gbps, representing a significant 89.4% improvement compared to the throughput (5.11 Gbps) of HS-PPO. Under heavy cloud, the throughput of RC-PPO maintains 7.06 Gbps, yielding a 51.8% improvement over the 4.65 Gbps of HS-PPO. The results demonstrate that the RC-PPO system exhibits substantial throughput improvement under light, moderate, and heavy cloud conditions. RC-PPO demonstrates the most pronounced throughput advantage over HS-PPO under moderate cloud-density conditions. In addition, under heavy cloud-density conditions (Figure 2c), a non-RL scheme is also evaluated, which follows a straight-line path from the starting point directly to the GS without any adaptive cloud avoidance. Results show that RC-PPO achieves the highest average throughput, outperforming both the non-RL RC and HS-PPO schemes. It confirms that the performance gain stems not only from the use of RCs but from the predictive trajectory optimization enabled by DRL.

**Figure 3:**
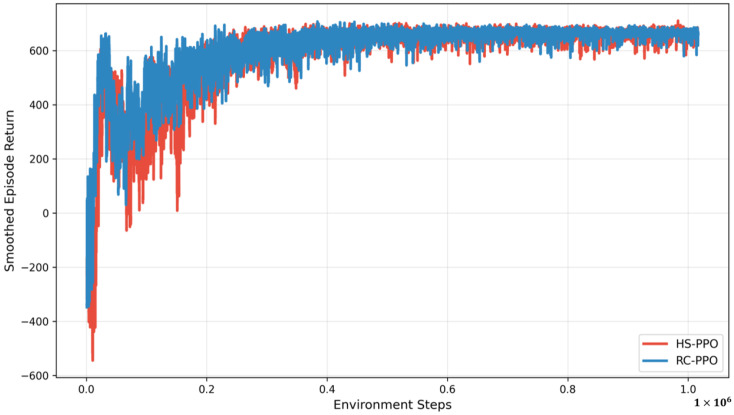
Training performance of HS-PPO and RC-PPO schemes.

Figure 3 illustrates the training performance of the RC-PPO and HS-PPO agents. Although a direct quantitative comparison of their absolute return values is complicated for the differences in reward design, the resulting HAPS trajectories suggest that both schemes achieve effective policy learning. Notably, despite the inherently sparser feedback of the RCs (informative feedback is available only upon successful decoding of entire data blocks), the RC-PPO agent exhibits slightly more stable convergence performance. This improved stability is attributed to a shaped-reward mechanism specifically designed to provide incremental feedback on packet decoding progress. By incorporating denser reward signals, RC-PPO effectively mitigates variance in policy gradient estimates, thereby improving sample efficiency throughout training.

## 5. Conclusions

This paper presents an integrated framework that combines RCs with a PPO-based DRL algorithm for HAPS trajectory optimization in a hybrid FSO/RF SAG system. By embedding potential-based reward shaping around decoding progress, the proposed RC-PPO method addresses key limitations of conventional HS schemes, enabling more reliable exploitation of both FSO and RF channels under stochastic cloud obstruction. Simulations demonstrate that RC-PPO improves achievable capacity and transmission reliability. Under moderate cloud scenarios, the average throughput of the RC-PPO agent is 9.68 Gbps, achieving 89.4% improvement compared to that of HS-PPO (5.11 Gbps). Moreover, RC-PPO exhibits more stable training convergence compared to HS-PPO.

The proposed framework in this paper can be further extended to multi-HAPS/multi-GS systems by adopting multi-agent reinforcement learning (MARL) strategies, such as Multi-Agent PPO (MA-PPO), where multiple HAPSs coordinate their trajectories and transmission policies to optimize coverage and load balancing.

## Figures and Tables

**Table 1:** Simulation parameters.

Parameter	Value
HAPS altitude ($H_{haps}$)	20 km
Clouds base altitude ($h_{0}$)	1 km
Clouds max altitude ($h_{\max}$)	10 km
Receiver aperture diameter (*D*)	1 m
Responsivity of PD (R)	0.8
Variance of background noise ($\sigma^{f}$)	250 μW
Noise power spectral density ($\sigma^{r}$)	−100 dB/MHz
Optical transmit power ($P_{t}^{f}$)	1 W
RF transmit power ($P_{t}^{r}$)	1 W
Telescope gain of transmitter, receiver ($G_{tx}^{f},G_{rx}^{f}$)	70 dB
Antenna gain of transmitter, receiver ($G_{tx}^{r},G_{rx}^{r}$)	50 dB
Optical wavelength ($\lambda^{f}$)	1550 nm
RF frequency ($f^{r}$)	30 GHz
Optical bandwidth ($B_{f}$)	10 GHz
RF bandwidth ($B_{r}$)	500 MHz

**Table 2:** PPO training hyperparameters.

Parameters	Values
Feature layers	[128, 64, 32] (ReLU)
Policy head init gain	0.01
Learning rate (α)	$3\times 10^{-4}$
Discount (γ)	0.99
Clip ratio ($\epsilon_{clip}$)	0.2
Entropy coeff./Value coeff.	0.01/0.5
Gradient clip	0.5
Mini-batch size	64
Update epochs per batch (K)	10
Total environment steps	$10^{6}$

**Table 3:** Final tuned reward weights and their tuning rationale.

Weight	Value	Role and Tuning Rationale
Throughput (α)	1	Fixed to establish reward scale, directly reflecting the objective of maximizing data rate.
Decoding Progress (β)	5	Provides dense gradients for decoding progress. Value chosen via grid search.
Heading Penalty ($\kappa_{\psi }$)	0.35	Penalizes abrupt heading changes. Initially small; increased if trajectories exhibited excessive jitter.
Distance Penalty ($\kappa_{d}$)	0.1	Encourages the agent to maintain proximity to the GS, accelerating learning convergence. Initially small; increased if the agent strayed too far.
Terminal Reward (*q*)	100	Task completion signal. Large scalar reward that reinforces successful mission completion.

## Data Availability

The original contributions presented in this study are included in the article. Further inquiries can be directed to the corresponding author.
